# Acceptability and feasibility of one-stop home-based genital self-sampling for female genital schistosomiasis, human papilloma virus and self-testing for trichomonas and HIV: the Zipime Weka Schista study in Zambia

**DOI:** 10.1186/s12905-026-04654-1

**Published:** 2026-07-09

**Authors:** Rhoda Ndubani, Olimpia Lamberti, Isaac Mshanga, Nkatya Kasese, Helen Kelly, Jennifer Fitzpatrick, Janet Chisaili, Ruth Mwanza, Melisa Choongo, Molen Ngoshe, Sandra Phiri, Georgina Mukombwe, Emily L. Webb, Helen Ayles, Kwame Shanaube, Amaya L. Bustinduy

**Affiliations:** 1 Zambart, Lusaka, Zambia; 2 Department of Clinical Research, London School of Hygiene & Tropical Medicine, London, UK; 3 University College Dublin, Dublin, Ireland; 4 Department of Infectious Disease Epidemiology, London School of Hygiene & Tropical Medicine, London, UK

**Keywords:** Female genital schistosomiasis, *Schistosoma haematobium*, Acceptability, Feasibility, Self-sampling, Women, Integration, Home, Clinic, HPV, Trichomonas, HIV, STI, Cervical cancer, Cervical precancer

## Abstract

**Background:**

Female genital schistosomiasis (FGS) is a neglected gynaecological disease that affects 56 million girls and women in sub-Saharan Africa. It is caused by the waterborne parasite Schistosoma (S.) haematobium and has been associated with HIV infection, with human papillomavirus (HPV) and cervical precancer. FGS alters the normal sexual and reproductive health of girls and women. Diagnosis and screening are bottlenecked, but previous studies have shown good acceptability of genital self-sampling at home for individual diagnosis of FGS, HPV and selected STIs. Here, we aim to determine the acceptability and feasibility of home-based multi-pathogen self-sampling and testing.

**Methods:**

The Zipime Weka Schista study is an ongoing longitudinal cohort integrating a one-stop home-based genital self-sampling for S. haematobium and HPV DNA detection with self-testing for HIV and Trichomonas vaginalis (Tv) in three communities in Zambia. Sexually active women aged 15-50 years were randomly selected by community health workers and visited at home where they were invited to provide two cervicovaginal self-swabs and a urine sample, and to conduct self-tests for HIV and Tv. During the home visit, community health workers collected information on the acceptability and feasibility of the multi-pathogen genital self-sampling approach using a questionnaire. A follow-up visit was done in clinic.

**Results:**

A total of 2,701 women were initially approached by community health workers and 2,532 were enrolled between January 2022 – March 2023. A total of 100% (2,532) women provided self-swabs, and 94.3% (2,389) and 55.4% (1,404) undertook Tv and HIV self-tests, respectively. Of these, 67% (1,694) were followed up in clinic. There was high acceptability 76.4% on the procedures for home multi-genital self-sampling. And many participants 87.2% preferred to be seen at home than in clinic. Some reasons stated were convenience 62.6%; privacy 47.9%; going to the clinic was not convenient 10.4%; lack of transport to go to the clinic 8.2%; unavailability due to work 4.6% and unavailability of childcare 2.7%.

**Conclusion:**

The study shows that home-based multi-pathogen self-sampling and testing is highly acceptable and feasible in three communities in Zambia. This has potential to increase access to diagnosis, treatment and care for different infections co-existing in women.

**Supplementary Information:**

The online version contains supplementary material available at https://doi.org/10.1186/s12905-026-04654-1.

## Introduction

Schistosomiasis is a parasitic disease acquired when people come into contact with larval forms of the trematode parasite of the *Schistosoma (S.)* genus, known as cercariae, which are released by freshwater snails acting as intermediate hosts [1]. Contact occurs while wading, bathing or washing in contaminated water, when cercariae emerging from their host snails penetrate a person’s skin and develop into mature worms, which can live within the blood vessels of the human host for years, sometimes for decades [2]. Human schistosomiasis affects around 200 million people worldwide annually and the majority of these infections (~ 165 million) occur in Sub-Saharan Africa [3]. It is estimated that approximately 30% to 75% of women with *S. haematobium* infection may develop genital lesions attributable to this infection [4]. The presence of *Schistosoma* ova in female reproductive organs is known as female genital schistosomiasis (FGS). The disease develops over a long period of time (months to years) from the time of infection, and most girls and women do not have access to treatment and care [5]. FGS has been described as one of the most neglected sexual and reproductive health (SRH) diseases in sub-Saharan Africa (SSA), with an estimated 56 million women and girls affected [6]. This can add to the global burden of other infections such as cervical cancer and HIV, which affects women disproportionately, and particularly young women in Sub-Saharan Africa [7].

Signs and symptoms associated with FGS include dyspareunia, bleeding after sex, vaginal discharge, abdominal and pelvic pain, sub-fertility and infertility and ectopic pregnancies which overlap with those seen in the most common sexually transmitted infections (STIs) [8]. One of the methods for FGS diagnosis is visual examination of the genital tract by colposcopy, identifying classic lesions including grainy sandy patches, homogenous yellow patches, abnormal blood vessels, and rubbery papules [9]. However, this method is costly and requires specialised equipment and training and is not always available in health facilities in endemic communities [10]. This method also lacks specificity and can be subjective with high degrees of non-agreement even between experts [11]. Further, there is a lack of awareness and recognition of FGS among health professionals as it is not part of standard medical training [6, 12, 13]. Therefore, FGS remains underreported, misdiagnosed and largely untreated [6, 14, 15].

FGS causes a high degree of inflammation in the genital tract which increases the risk of acquisition of STIs and HIV [16, 17]. As a result, women and girls with FGS are up to three times more likely to acquire HIV [18–20]. FGS has been associated with high-risk HPV genotypes 16/18/45, indicating that FGS may increase susceptibility to, or persistence of oncogenic HPV [21]. This increased risk of HPV in women with FGS could potentially lead to higher risk of cervical precancer [6]. Moreover, cervical cancer and *S. haematobium* eggs have been reported together in histopathology specimens of women with cervical precancer [22]. These findings are important in sub-Saharan Africa, where both *S. haematobium* and cervical cancer rates are high. Recent studies have reported on the growing suspicion that hybrid forms of *S. haematobium* with *S. mansoni* and *S. mattheei* and *S. bovis* could be involved in FGS and, therefore, that not only *S. haematobium* is involved. However, more research is needed to understand its health implications [6].

Considering the potential overlap between FGS, HIV, HPV and cervical cancer and increased prevalence of these infections, adopting an integrated approach with comprehensive screening and broader symptomatic and clinical management of sexual and reproductive health (SRH) programs for these infections, offers an opportunity to reach more girls and women [6, 23, 24] and provides an important first step for diagnosis, treatment and prevention of these infections. The World Health Organisation (WHO) neglected Tropical Diseases roadmap 2021–2030 also emphasizes the need for integration of disease interventions to strengthen the health systems and improve outcomes for affected populations [15]. Accurate and effective diagnosis and treatment of FGS can prevent unnecessary cervical cancer treatments, reduce misdiagnosis of STIs, avert onward HIV transmission, and reduce repeated healthcare visits, ultimately reducing strain on the health system [25].

Closer to the user and low-cost screening tools for multi-pathogen detection could be an effective intervention for service integration [15]. Several studies have highlighted the importance of integrating disease or sector-specific interventions into broader health services to improve health coverage and efficiency [24]. The current shift from clinic-based to community-based screening and testing for cervical cancer and HIV across SSA provides a further opportunity to deliver an integrated home-based screening and testing package that includes FGS testing [6, 26]. In community-based fully integrated models, an FGS and HPV screening package could be included in HIV self-testing kits delivered at home by a community-health worker [27]. This would empower women with a one-stop screening and testing approach at home. This could represent a cost-effective strategy to detect multiple pathogens and increase surveillance for different SRH conditions [10].

Integrating FGS and SRH services is potentially associated with reduced costs and increased efficiency compared to delivering screening separately [28, 29]. From a patient’s perspective, integrating services, delivered either at home or in clinic, can reduce the time and inconvenience of utilizing healthcare for multiple conditions, thus improving patient’s access to care and health outcomes [30]. From a provider perspective, integration improves processes and resource allocation by capitalizing on commonalities in care delivery and distribution systems and by sharing capital resources [26, 30]. However, for these interventions to be deployable, their acceptability and feasibility need to be ascertained.

Several studies have assessed the acceptability and feasibility of self-sampling for HPV and STIs and self-testing for HIV [31–35] and more recently for FGS [36, 37]. To date, no study has assessed the acceptability and a feasibility of a multi-pathogen genital self-sampling and testing done at a single visit at home. The aim of the Zipime Weka Schista study [38] was to develop a holistic approach for the community-based diagnosis of FGS through a comprehensive package for SRH screening including HPV, STIs & HIV. Here, we aim to determine the acceptability and feasibility of this approach in a large ongoing cohort in Zambia.

## Methods

### Study design and population

The Zipime Weka Schista study is an ongoing (2021–2028) longitudinal cohort integrating home-based genital self-sampling for detection of FGS, high-risk Human Papillomavirus (HR-HPV), and self-testing for *Trichomonas vaginalis* (*Tv)* and HIV in three communities in Zambia. Methods of the study are described elsewhere [38]. Briefly, sexually active girls and women aged 15–50 years and not pregnant, were randomly selected to take part in the study. Schista community workers (SCWs) recruited women during a home visit and obtained two cervicovaginal self-swabs and a urine sample and offered self-tests for HIV and *Tv*. Household demographic and symptom questionnaires were also administered [36]. Women were referred to the clinic where a midwife collected genital samples and obtained images using hand-held colposcopy. Here, we present cross-sectional baseline data collected between January 2022 and March 2023.

### Sampling strategy

Community-based cluster sampling was used to obtain a random sample of participants from the study communities. Communities and their households were mapped, and each community was subdivided into blocks of approximately 50 households and all eligible women aged 15–50 years invited to take part. Blocks were randomly selected using random number generation in Stata and were being visited sequentially in the order that they were randomly selected. Randomization was done using Stata 14.0 (StataCorp, LP, College Station, US).

### Home visit and questionnaire

SCWs conducted home visits and study procedures were conducted in a private area of the house. Study information was given in a language of the participant’s choice, along with FGS education using a World Health Organization’s “Female Genital Schistosomiasis Pocket Atlas” [39]. Study information and study procedures were also explained in detail.

Participants were shown self-sampling procedures with the use of a three-dimensional model of a female anatomy and a test swab for illustration. Photos in the World Health Organization’s “Female Genital Schistosomiasis Pocket Atlas” were also displayed as a visual aid [39]. Thereafter, participants were asked to self-sample with the two given swabs in a private room and later placed these swabs inside 2 ml tube of PrimeStore^®^ MTM molecular transport media (donated by Longhorn Vaccines and Diagnostics LLC, Bethesda, MD). The two genital self-swabs were then transported and analysed in the laboratory by trained laboratory technicians. The genital self-swabs were analysed for *Sh* by Polymerase Chain Reaction (PCR) and HPV by rDNA by GeneXpert. A genital self-swab for *Trichomonas vaginalis (Tv)* was also performed during the visit. Results were read by the SCW and given out immediately. If positive, the participant was referred to the clinic to meet with the study midwife and was given treatment. A 10- 30 ml sample of urine was also collected. The urine test was analysed via microscopy for *Sh* ova detection and circulating anodic antigen (CAA). These tests were done in the laboratory, and results were shared back to the participants.

In addition, SCWs also offered one oral (saliva) for HIV self-test preceded by counselling [35]. Instructions were read out to the participants before collecting the test. The participant proceeded with the oral swab in the presence of the SCW. If the results were positive, a confirmatory test was done immediately, if the confirmatory test was also positive, the woman was then referred to the local clinic for further HIV management and linkage to care. We did not record any refusals from participants to provide all specimens.

Participants then completed a questionnaire, with responses captured on hand-held tablets. The questi`onnaire assessed basic demographics, information regarding genital symptoms, sexual behaviour and the participant’s assessment of the acceptability of self-sampling and testing, through their responses to 15 questions each measured on a five-point Likert scale (Test S1) [36, 40, 41]. The SCW then asked the participant to complete further study procedure at the local clinic at their earliest convenience. A study identification card with the unique identifier number was placed in a badge and handed over to the participant to carry to the clinic where a midwife conducted the study visit. The study midwives obtained two cervicovaginal swabs and collected images using a handheld colposcope.

### Ethics and informed consent

SCWs obtained written consent from all eligible participants during the home visit. Participants who were unable to provide written informed consent were recruited in the presence of a witness with the participant placing their thumbprint on the consent form. All study staff underwent training in research ethics and confidentiality. The study was approved by the University of Zambia Biomedical Research Ethics Committee (reference number: 1858–2021), the Zambia National Health Research Authority (reference number: NHRA00012) and the London School of Hygiene and Tropical Medicine research ethics committee (reference number: 25258). Permission to conduct the study was further given by Ministry of Health, the Provincial and District Health offices. Identifiable data collected is stored securely and their confidentiality protected in accordance with the Data Protection Act 1998.

### Data management and statistical methods

Participant data were entered using Open Data Kit (ODK) collected on tablet devices [42]. All field and clinic data were entered in the ODK system and uploaded to a secure server in Zambia. Acceptability was assessed by the following outcomes: the proportion of women who rated home based self-sampling and testing to be “easy” or “very easy” (for each of urine, vaginal, cervical self-sampling and oral self-test), the proportion who were willing to self-sample again “in the same setting” (for each of urine, vaginal, cervical self-sampling and oral self-test), and the proportion who would prefer to “sample at home” (versus sampling at the clinic). Proportion who found the instructions for genital (cervicovaginal) self-sampling to be “easy” or “very easy”.). Continuous variables were summarized by median and interquartile range (IQR), and categorical variables by frequency and percentage. The women could choose among four possible reasons to explain why they preferred either the home-based self-sampling and testing or clinic-based sampling (1) convenience (2) privacy (3) going to the clinic was not convenient (4) lack of transport to go to the clinic (5) unavailability due to work (6) unavailability of childcare To evaluate the data on Preference (PREF) and Ease of Self-Sampling, we conducted a bivariate analysis using Pearson’s Chi-square (x2 ) tests for independence. We used nominal categorical of geographic study sites, binary categorical variable indicating preferred screening location. And ordinal categorical variable of self-sampling. Cross tabulation generated contingency tables to observe how many individuals in each district selected each response category. Pearson’s Chi-square was used to determine if the distribution of responses was independent of district. Significance Threshold: α = 0.05. Stata output showed (Pr) = 0.000, interpreted as *p* < 0.001. Since this is below 0.05, the results are statistically significant, indicating that participants’ preferences and ease of sampling were significantly associated with their district. Data was analysed using Stata 14.0 (StataCorp, LP, College Station, US).

## Results

**Fig. 1:**
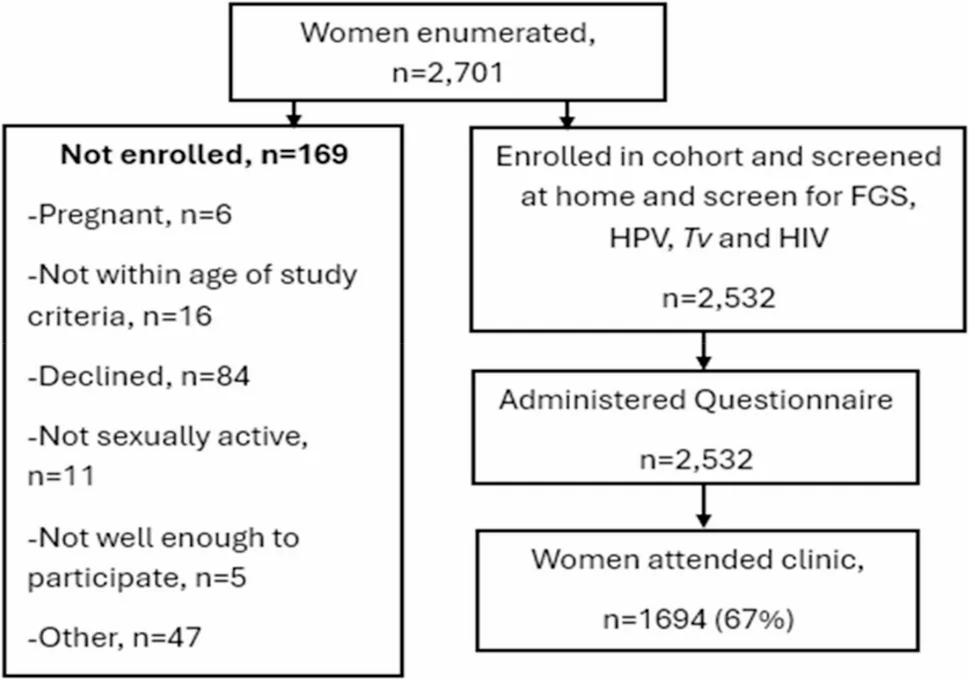
Zipime-Weka-Schista study flow diagram for the recruitment and enrolment of participants at baseline

A total of 2,532 out of the 2,701 women contacted (93.7%) were eligible, enrolled and completed the initial home-based visit with the SCWs. Of the 6.2% of women (169 who were not enrolled into the study, 3.5% were pregnant, 9.4% were outside age criteria, 49.4% reported that they were not interested in taking part in the study, 7.6% had a family member who declined for them, 3.0% were not well enough to participate,6.5% had not had their sexual debut, 10.0% were too busy and could not make time for study procedures, 6% had concerns about certain study components and 1.2% were not informed prior to the visit by the research team (Fig. 1).

The median age was 28 years (IQR 22–36). Approximately half of the total recruited participants 53.1% had obtained some secondary school education. 79.5% were not in formal employment (Table 1). Of the 2,532 participants completing the home-based visit, 1,694 (67%), completed the clinic visit (Fig. 1).

**Table 1:**
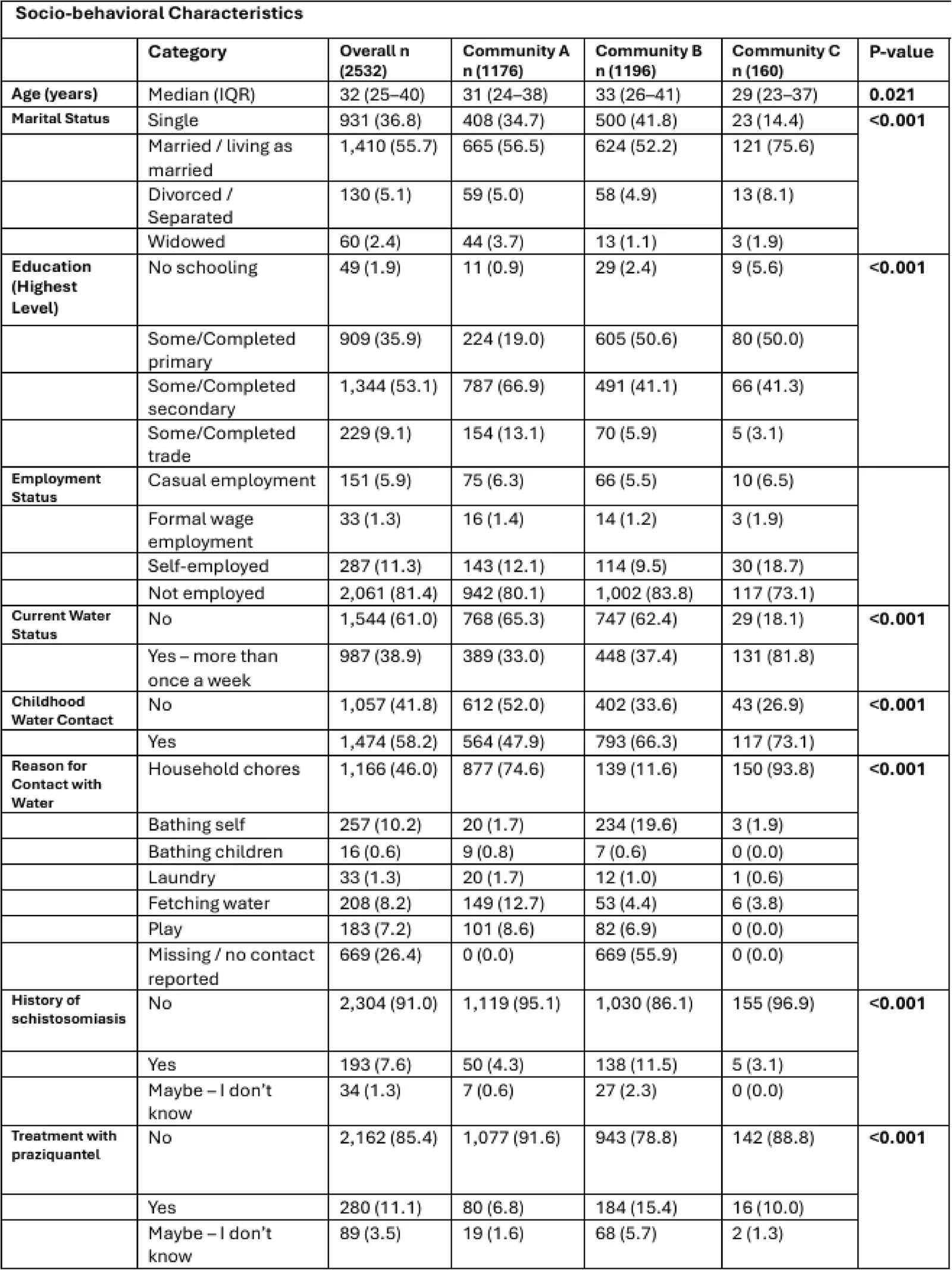
Baseline characteristics of 2,532 Zambian women living in Schistosoma haematobium endemic areas in Zambia. Comparison of community A, B and C

### Acceptability and feasibility of procedures

**Fig. 2:**
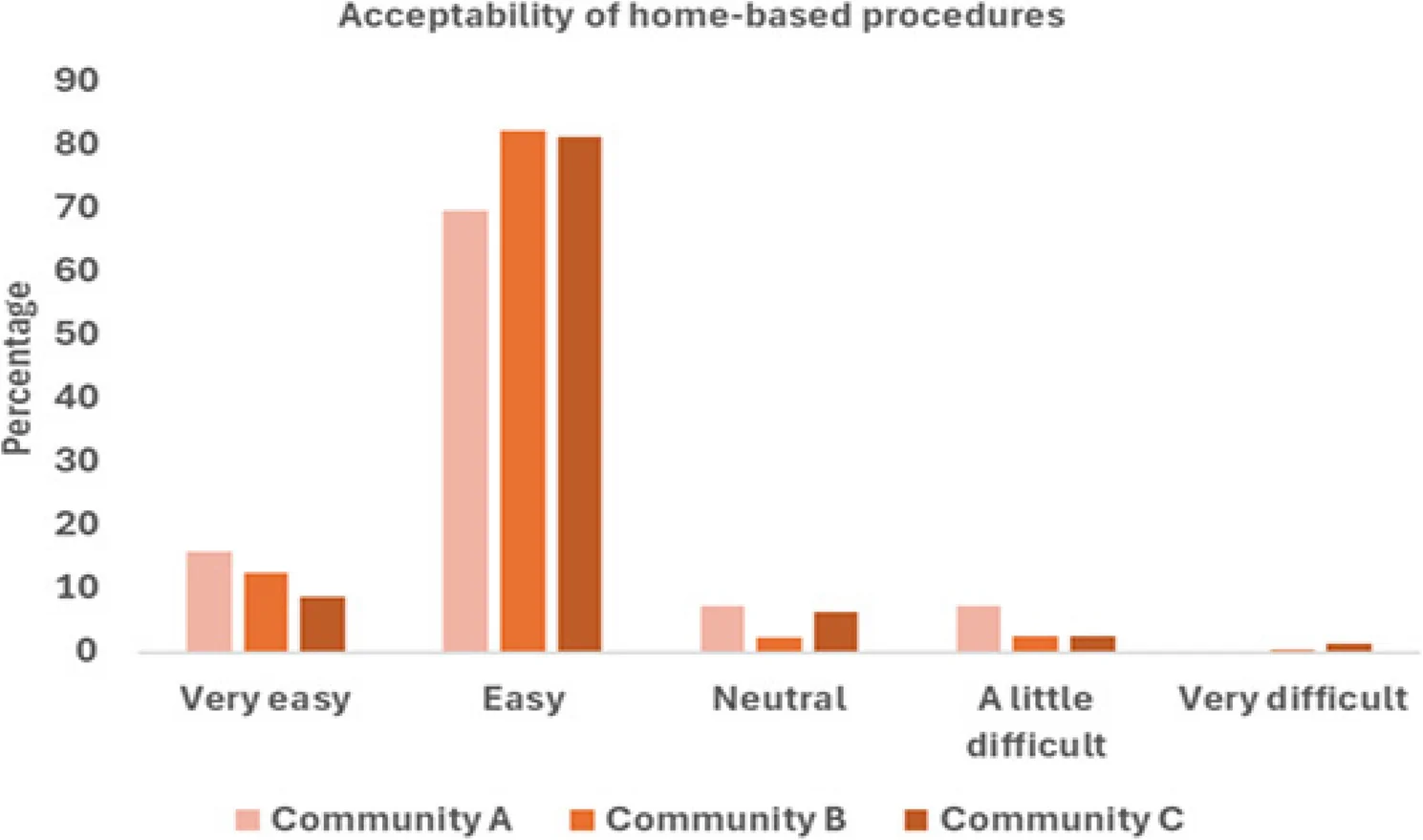
Responses on ease of self-sampling and testing of the 2,532 women at baseline.

As shown in Table 2 and Fig. 2 a high proportion of women in the study indicated that self-collection of the genital specimen for FGS, HPV, and self-testing for *Trichomonas vaginalis* and for HIV was “very *easy*” (13.8%) or “*easy*” (76.4%) on a 5-point Likert scale. Most participants indicated that they would be willing to self-sample and self-test in the same setting again (99.5%). Notably, only 4.7% and 0.2%, found it “*a little difficult”* and “*very difficult”* to self-collect the genital specimens. Overall, most women preferred to collect the specimens at home (87.2%, compared with clinic-based sampling (12.8%) (Table 2). These results were consistent across communities, with women from all three communities reporting a preference to self-collect specimens from home (Community A: 80.9%, 951/1176; Community B: 95.7%, 1,144/1,196; Community C: 70.6%, 113/160) compared to attending the health facility. Some reasons for the preference stated were that it is more convenient 71.8% (1585) there was more privacy at home 1215 (55.0%); going to the clinic was not convenient 12.0% (264) lack of transport to go to the clinic 9.4% (208) unavailability due to work 5.3% (118) and lack of childcare, 3.1% (69). Further, there were differences between settings in terms of preferences.

**Table 2:**
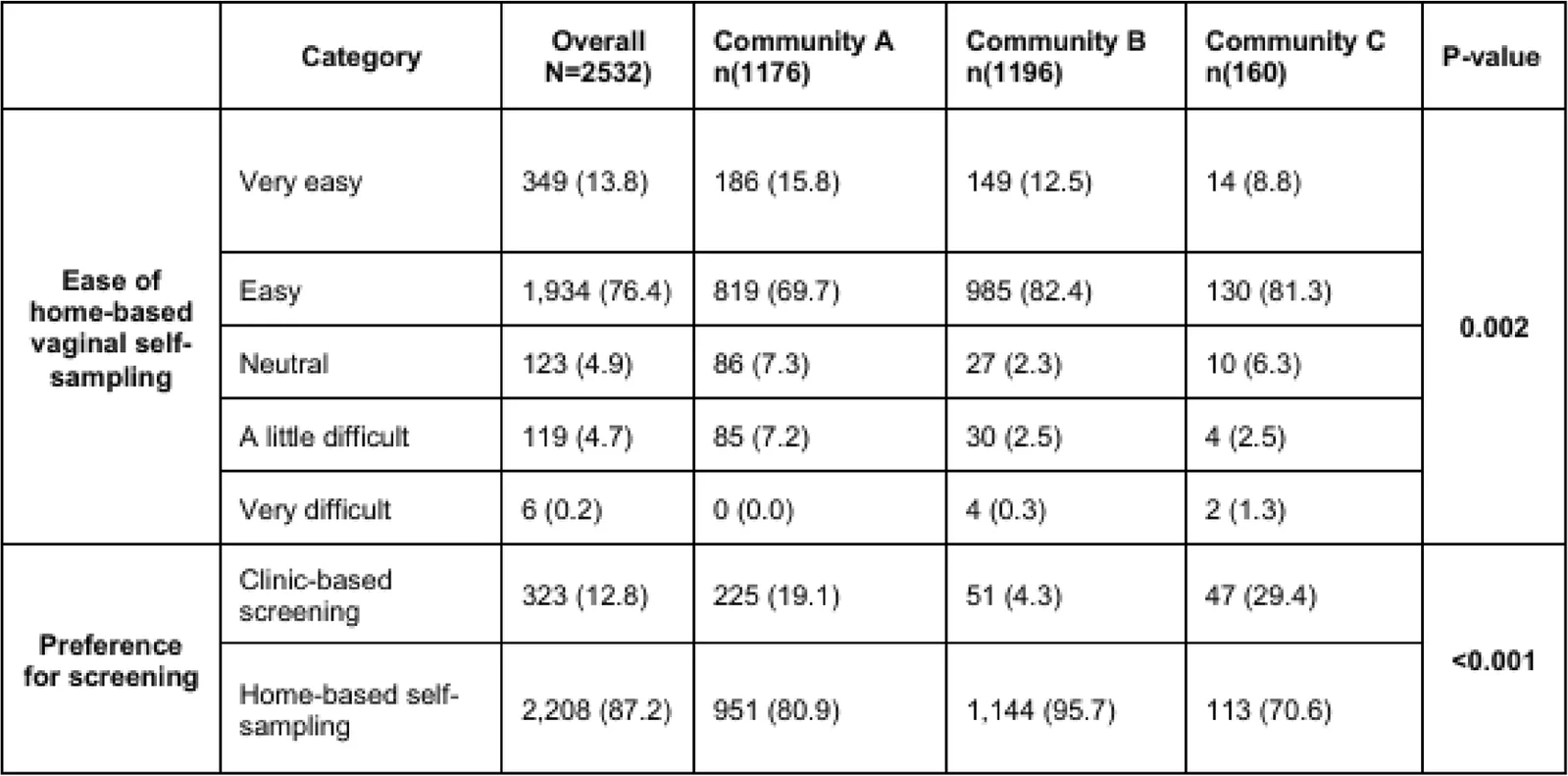
Acceptability of self-sampling and preference of screening by study participants

There was little evidence that age, marital status and employment status were associated with a participant’s preference. Given that the preference for self-sampling was universal across the groups examined in the crude analysis, multivariable analysis was not performed.

## Discussion

In this study, we found that self-sampling for the diagnosis of FGS, HPV and self-testing for HIV and *Trichomonas Vaginalis* has high acceptability and feasibility among women aged 15 to 50 years enrolled in the Zipime Weka Schista cohort study in Zambia. All women participating in the study provided all three self-collected specimens (oral, vaginal and cervical), and a high proportion found self-sampling “easy”.

Our study is in agreement with other studies in which individual genital self-sampling for either FGS, HPV or STIs and self-test for HIV has been acceptable among young and older women of different ethnicity, educational and socio-economic status [7, 32, 35, 36]. A cross-sectional study conducted in Zambia also found high (86.6%) acceptability and feasibility of the genital self-sampling for the diagnosis of FGS in young women aged 18–36 years [36]. Another study conducted in Zambia highlighted the high prevalence of FGS in adult women living in urban areas [43] which showed the importance of screening for FGS in Zambia.

The integration of FGS services into existing healthcare programmes presents a significant opportunity to improve the control of FGS and address the neglect of this condition [6]. Researchers and policymakers recognize the importance of integrating health services to optimize resource utilization and improve healthcare outcomes. To ensure the effective implementation of new FGS screening strategies in *S. haematobium* endemic communities, there is a need to evaluate the available capacity within the health system. Economic evaluations of cervical cancer screening have shown that community-based genital self-sampling followed by molecular testing is a feasible and cost-effective option, either as an addition to existing screening programmes or as a primary screening strategy [44]. However, successful implementation of such models requires additional resources and capacity building within the health system. This makes the implementation rare in practice due to excessive costs and healthcare budget constraints [27, 45].

Genital self-sampling and self-testing as an alternative to clinician collected samples, has been validated in recent years for the diagnosis of different genital tract infections [37, 46]. This approach has shown to increase adherence among under-screened women, those who may not visit the health facility regularly and women who may be geographically isolated [40, 47]. In addition, self-sampling has been shown to be cost effective, highly acceptable in terms of ease to use, convenience, privacy, physical and emotional comfort and less embarrassing [47–49]. Similarly, in this study, the home-based approach substantially reached women who were at risk of infection, had low awareness and were first -time testers. This has potential to reduce the burden and vulnerability for FGS, HIV, *Tv*, HPV and ultimately cervical cancer. While there have been calls for integrated FGS in SRH programmes within the health systems [23, 50], integration encompasses managing and delivering health services in a comprehensive and coordinated way, tailored to patients’ needs whilst ensuring continuity of care across disease programmes and at different levels of the healthcare system [26], our study is the first to show the acceptability and feasibility of integrating screening of FGS with HPV, Tv and HIV at household level and in limited resource settings. Further, in this study, feasibility is defined by high rates of participant enrolment and procedural completion. The strong conversion rate from approached candidates (*n* = 2,701) to enrolled participants (*n* = 2,532) demonstrates the program’s reach and practical viability. Additionally, the 100% completion rate for self-swabbing confirms that the primary task is highly feasible for the target population.

The ease of self-sampling for the home-based procedures in this study was reported as very easy (13.8) and easy (76.4%) by the majority of women recruited into the study (Table 2). Some of the reasons given were the very clear instructions and the aid of the 3-Dimensional model, which helped them visualize the technical instructions. These findings show high feasibility and suggest that self-sampling could be a viable option for wider implementation and for policy decision and show potential for consideration of integrating self-sampling into testing programs. However, some participants reported that they found the self-sampling a little difficult (4.7%) and (0.2%) very difficult (Table 2), this could indicate that more additional resources such as time and demos could be needed for certain groups of women. However, most women indicated that they would be willing to self-sample in the same setting again. Our findings show that home-based self-sampling proves to be a convenient and comfortable method of screening in such settings and has potential to increase uptake.

A qualitative study conducted at the cervical cancer screening clinic at ORCI in Dar es Salaam, showed that most participants liked self-sampling and still preferred it over provider-based sampling despite experiencing some difficulties with the procedure. They needed a health provider present when conducting the self-sampling [31]. In other studies, health providers prepared the women prior to the self-sampling by providing health education, allowed the women to touch the swab and provided support through-out the process because some women were worried about the accuracy of the test. A cross-sectional study conducted in Hong Kong in women aged 25–35 and 45_> that explored the feasibility and acceptability of HPV self-sampling found high acceptability (80%) of self-sampling in under screened and never-screened women [40].

Women from all three study communities preferred to self-collect specimens from home compared with clinic-based provider sampling. If adopted widely as a screening procedure, this preference may increase the uptake of screening for multiple infections. Further, women reported reassurance of having a community health worker who explained the procedures using the three Dimension model making it easy to follow the instructions and take the samples to a laboratory facility. The community health workers also provided knowledge on the different genital infections that were being screened for, this could have increased the acceptability levels. Community health workers can play a crucial role in raising awareness about the different infections, mostly in communities with low awareness for diseases like FGS and HPV and potentially increase screening [33]. Our study has demonstrated feasibility through the work of community health workers who were able to successfully provide clear instructions for self-sampling and testing to the women and increased uptake to the home-based integrated screening and testing for FGS, HPV, HIV and *Tv.*

In this study, we also found that women preferred to self-sample at home because it was more convenient, there was more privacy. They further reported that going to the clinic was not convenient, there was lack of transport and difficulty reaching clinic due to work, and lack of childcare. Other studies have also highlighted stigma, travel time and waiting time at the clinic as further hurdles to this approach [51]. Our study reports that 67% of the women and girls seen at home completed the clinic visits, the study has shown the ability to transition 67% of participants from home sampling to a clinic visit demonstrates the feasibility of linking community-based screening to formal medical care.

As previously reported, high acceptability of the procedures was associated with higher education levels and those who live in distant areas [40, 52, 53]. This may indicate that women with a higher education may be more confident with self-sampling than women with lower education and may have a better understanding for choice of screening methods [54]. Another study found that religion significantly determined knowledge and prevention behaviour that influenced decision in the uptake of HPV self-sampling [40]. It would be important to put all these factors into consideration during implementation to increase screening uptake. However, the high acceptability (76.4%) and stated preference for home visits (87.2%) in this study, strongly indicate that the home-based delivery model is the most viable strategy for reaching this population.

### Limitations

Although, the home-based self-sampling and testing strategy has shown to be acceptable and feasible, there is need to investigate the cost effectiveness of this strategy which is going to support evidence-based policy of integrating screening even at national level. The participants were interviewed in their homes on questions about preference of screening between home- and clinic-based screening, this could have created a bias and influenced their responses. We did not ask about preference during the clinic visits. The reasons for acceptability were not explored in detail. Therefore, more qualitative studies will need to be conducted to explore how these factors influence choice of screening methods.

## Conclusion

Integrated self-sampling and testing procedures for multi-pathogen detection were highly acceptable and feasible in women aged 15–50 years in three communities in Zambia, with most women finding it easy and expressing willingness to use it again. Home-based procedures were preferred by most of the participants over clinic-based procedures. Our study provides new evidence on acceptability and feasibility on integration of a home-based multi-pathogen self-sampling and testing methods. This represents an evidence-based novel direction for national policy to increase access to diagnosis, treatment and care for different infections co-existing in women of childbearing age.

## Supplementary Information

Supplementary Material 1.

## Data Availability

Data are available upon reasonable request. Please contact the corresponding author if required.
