# Supplementary material for: Acceptability and feasibility of one-stop home-based genital self-sampling for female genital schistosomiasis, human papilloma virus and self-testing for trichomonas and HIV: the Zipime Weka Schista study in Zambia
Source: BMC Womens Health. 2026 Jul 9;26:494. doi: 10.1186/s12905-026-04654-1 (PMC13632438; doi:10.1186/s12905-026-04654-1)
Supplement: Supplementary file 1 [file 12905_2026_4654_MOESM1_ESM.docx]

**Supplementary material for “Acceptability and feasibility of one-stop home-based genital self-sampling for Female Genital Schistosomiasis, Human Papilloma Virus and self-testing for Trichomonas and HIV: The Zipime Weka Schista Study in Zambia”**

**Text S1. Questionnaire for Zipime Weka Schista Study**

**For the following three questions, please use the scale to rate your experience with sampling at home.**

**The scale is 0 to 4 (0 is “very easy” and 4 is “very difficult”). On a scale of 0-4, how would you describe your experience with self-sampling?**

| **0** | **1** | **2** | **3** | **4** |
| --- | --- | --- | --- | --- |

**Very Easy	Easy	Neutral	A little difficulty	Very difficulty**

**Number	Question**

1 I found genital self-sampling to be:
2 I found cervical self-sampling to be:
3 I found the instructions for genital (cervicovaginal) self-sampling to be:
4 I found the instructions for taking a self-sample to be:

**For the following questions, please use the scale to rate your experience with sampling at home. For the following questions, the scale is 0 to 4 (0 is a strong yes and 4 is a strong no):**

| **0** | **1** | **2** | **3** | **4** |
| --- | --- | --- | --- | --- |

**Strong Yes	Yes	Maybe	No	Strong No**

**Number	Question**

5 I would be willing to take a vaginal self-sample in the future:
6 I would be willing to take a cervical self-sample in the future:
7 I would recommend self-sampling to my friends:
8 Self-collecting a vaginal swab was painless:
9 Self-collecting a cervical swab was painless:
10 I am confident I collected the specimens properly:
11 I am confident I collected a sample from my vagina:
12 I feel confident I collected a sample from my cervix:

Do you prefer to take your samples at home, or would you prefer to take samples at the clinic?

**Number	Question**	**Responses**

13 I prefer doing sample at home because:	0 = Home 1=Clinic
14 I prefer having samples performed in the clinic:
15 I would self-sample again in the same setting:

For question 13: I prefer doing the sampling at home because. Choose all that apply.

**Coded answer:**

0 = it is more convenient

1 = I don’t have transportation to go to the clinic

2 = I don’t have childcare to go to the clinic

3 = I need to work and can’t go to the clinic

4 = I have more privacy at home

5 = Going to the clinic was not convenient

6 = Other
